# Minimal Invasive Abdominal Sacral Colpopexy and Abdominal Lateral Suspension: A Prospective, Open-Label, Multicenter, Non-Inferiority Trial

**DOI:** 10.3390/jcm12082926

**Published:** 2023-04-18

**Authors:** Eleonora Russo, Maria Magdalena Montt Guevara, Koray Gorkem Sacinti, Giulia Misasi, Maria Falcone, Riccardo Morganti, Liliana Mereu, Francesca Dalprà, Saverio Tateo, Tommaso Simoncini

**Affiliations:** 1 Division of Obstetrics and Gynecology, Department of Experimental and Clinical Medicine, University of Pisa, 56126 Pisa, Italy; 2 Department of Obstetrics and Gynecology, Ankara University School of Medicine, 06100 Ankara, Turkey; 3 SOD Clinical Trial Statistical Support, Azienda Ospedaliero Universitaria Pisana, 56126 Pisa, Italy; 4 Department of Provincial Health Services, Local Health of Trento, 38123 Trento, Italy; 5 Department of Obstetrics and Gynecology, Santorso Hospital, 36014 Vicenza, Italy; 6 Department of Obstetrics and Gynecology, Centre Hospitalier de Troyes, 10003 Troyes, France

**Keywords:** apical defect, minimally invasive abdominal surgery, lateral suspension, sacral suspension

## Abstract

Background: Abdominal minimally invasive surgery has become increasingly prominent for the treatment of prolapse. Abdominal sacral colpopexy (ASC) is the gold standard for the treatment of advanced apical prolapse; however, alternative surgical approaches such as the abdominal lateral suspension (ALS) have been developed to improve patient outcomes. This study aims to determine whether ALS improves outcomes compared to ASC in multicompartmental prolapse patients. Methods: A prospective, open-label, multicenter, non-inferiority trial was conducted in 360 patients who underwent ASC or ALS for the treatment of apical prolapse. The primary outcome was anatomical and symptomatic cure of the apical compartment at 1-year follow-up; secondary outcomes included prolapse recurrence, re-operation rate, and post-operative complications. A 300-patient cohort was subdivided into 200-patients who underwent ALS and 100-patients who underwent ASC. The confidence interval method was used to calculate the *p*-value of non-inferiority. Results: At the 12-months follow-up, the objective cure rate of the apical defect was 92% for ALS and 94% for ASC (recurrence rates were 8% and 6%, respectively, and the *p*-value for non-inferiority was <0.01). The mMesh complication rates were 1% and 2% for ALS and ASC, respectively. Conclusions: This study demonstrated that the ALS technique is not inferior to the gold standard ASC for the surgical treatment of apical prolapse.

## 1. Introduction

Pelvic organ prolapse (POP) is a common distressful condition, affecting women around the world and its prevalence ranges from 3 to 56% [1,2]. Advanced prolapse with apical failure is the most challenging situation with several surgical treatment options [3]. Abdominal minimally invasive surgery (MIS) has become widely used for advanced prolapse treatment and the access to robotic assistance has enabled the transition of many procedures from an open to a minimally invasive technique [4]. 

Mini-invasive abdominal sacral colpopexy (ASC) is widely regarded as the gold standard for the treatment of advanced apical prolapse [3]. Nevertheless, laparoscopic ASC is a complicated technique requiring sophisticated surgical skills and minimizes the risk of significant consequences, most notably sacral dissection. This fact has resulted in the development of innovative abdominal methods [5]. 

Abdominal lateral suspension (ALS) is a surgical procedure that has been proposed to repair apical and anterior prolapse without the need for sacral promontory dissection. The anterior vaginal wall, uterine cervix, and isthmus are sutured to a T-shaped synthetic mesh graft implanted in the vesicovaginal septum during ALS. The mesh is attached bilaterally to the abdominal wall, posterior to the anterior superior iliac spine, through the lateral arms. The technique enables concurrent treatment of apical and anterior POP. 

The current studies on laparoscopic lateral suspension (LLS) have demonstrated a success rate of greater than 90% in patients with anterior and apical prolapse, comparable to the laparoscopic sacrocolpopexy (LSC) [6,7,8,9,10,11]. 

There are limited reported studies on robotic lateral suspension (RALS) [12,13]. Over a two-year follow-up interval, we recently demonstrated that RALS has an objective cure rate of greater than 90% for advanced apical and anterior POP [14].

The comparative efficacy of ALS and ASC on apical repair has not been assessed and presently no studies have conducted a non-inferiority trial comparing these two procedures. 

The purpose of this study is to describe a prospective, open-label, multicenter, non-inferiority trial that compares the gold standard treatment for apical prolapse (ASC) to a recent ALS surgical technique.

## 2. Materials and Methods

### 2.1. Study Design and Participants

This is prospective, open-label, multicenter, non-inferiority trial. We recruited 360 patients with advanced apical prolapse who underwent ASC or ALS. It was conducted at the University Hospital of Pisa and at the Gynecologic Department of Santa Chiara in Trento between April 2013 and January 2019. 

Three hundred patients were required to investigate the non-inferiority condition, with an ALS/ASC ratio of 2. We applied a propensity score method to homogenize the patient sample on body mass index (BMI) and age, and we eliminated 60 patients who would affect the distribution. The 300-patient cohort was subdivided into 200 patients who underwent ALS and 100 patients who underwent ASC. 

The inclusion criteria were symptomatic multi-compartmental prolapse with apical failure, negative cervical cytology, and no abnormal uterine bleeding. Patients with contraindications for laparoscopic surgery, known malignancy of the cervix or abnormal uterine bleeding, and women who were reluctant to return for follow-up were excluded from this study. 

Patients eligible for this study were counseled about the duration of follow-up required for this study and gave their written informed consent in accordance with the Declaration of Helsinki. The study was carried out under the recommendations of the Good Clinical Practice (ICH/GCP). 

### 2.2. Clinical Assessment and Procedures

A preoperative clinical examination and POP-related symptoms assessment was performed at baseline. When patients presented with clinically symptomatic anterior and apical prolapse POP-Q stage ≥ II, they were offered ALS. Patients were offered supracervical hysterectomy in the presence of uterus abnormalities (e.g., leiomyomas, adenomyosis, endometrial abnormalities). In all other cases, we performed a uterus-preserving ALS approach. Patients were offered the ASC procedure when they presented with multi-compartment POP, including anterior, apical, and posterior defects (e.g., enterocele and/or high rectocele) POP-Q stage ≥ II. In the ASC procedure, a supracervical hysterectomy was performed when the uterus was present. 

ALS and ASC were performed by the standard laparoscopy and robotic approach. We used a T-shaped tetanized polypropylene mesh graft (TiLOOP “Prof. Dubuisson” 9 cm × 41.5 cm, 65 g/m^2^) for the ALS surgery. ASC was performed using a Y-shaped polypropylene graft (Alyte 27 cm × 5 cm, <20 mg/m^2^ Bard Inc., Covington, GA, USA or Restorelle 27 cm × 4 cm, 20 mg/m^2^ Coloplast Corp., Minneapolis, MN, USA).

A systematic post-operative assessment was performed at 6 and 12 months, including the POP-Q stage measurement and targeted questions about subjective satisfaction. 

### 2.3. Outcome Measures

The primary surgical outcome was the subjective and objective cure for apical POP at 12 months. The anatomic objective cure was considered as POP-Q ≤ 1. The subjective cure was considered the absence of vaginal bulge post-operatively. The secondary outcomes included recurrence of prolapse, reoperation rate, and post-operative complications. 

We followed the International Urogynecological Association’s reporting guidelines for surgical outcomes [15]. According to the Clavien–Dindo system, complications were classified and the mesh-related complications were proposed by the joint International Urogynecological Association/International Continence Society (IUGA/ICS) complication classification [16,17,18].

### 2.4. Statistical Analysis

The condition of non-inferiority for ALS surgery compared to ASC surgery (defined as a prolapse recurrence variation rate of less than 10%) was analyzed. The number of patients required to investigate the non-inferiority condition was 300. Three hundred patients with an ALS/ASC ratio of 2 were selected using a propensity score method adjusted for age and BMI from a sample of 360 patients. Absolute and relative frequency was applied to categorical data, whereas mean and standard deviation were calculated for continuous data. A Chi-square test and *t*-test (two-tailed) were used to analyze categorical and continuous data, respectively. The confidence interval method was applied for determining the non-inferiority *p*-value. SPSS v.27 technology carried out the statistical analysis; *p* values < 0.05 were considered statistically significant.

## 3. Results

We involved 360 patients (60 patients were excluded according to BMI and age distributions) from two Italian centers who had ALS or ASC with symptomatic stage II–IV apical prolapse. Table 1 summarizes the baseline demographic characteristics, POP-related symptoms, and surgical history. A total of 200 patients were assigned to ALS and 100 to ASC; both groups were well-balanced.

Overall, ALS and ASC were performed using a laparoscopic approach in 36 (18%) and 13 (13%) cases, and with robotic assistance in 164 (82%) and 87 (87%) cases, respectively. In the ALS group, 187 (93.5%) patients underwent uterus-preserving surgery, while 6 (3%) patients had a subtotal hysterectomy; 7 (3.5%) patients underwent ALS on the vaginal vault. In the ASC group, 33 (33%) patients received a uterus-preserving surgery and 33 (33%) underwent a subtotal hysterectomy; 34 (34%) patients had ASC on the vaginal vault (Table 1 and Table 2).

The mean operating time was 123 ± 33 min and 193 ± 55.6 min for ALS and ASC, respectively. The mean length of hospitalization following surgery was two days in both groups. There were no significant intraoperative complications or conversions to laparotomy (Table 2).

### 3.1. Primary and Secondary Outcomes

#### 3.1.1. Objective and Subjective Cure

After the 12-month follow-up, the objective cure rate for the apical defect was 92% for ALS and 94% for ASC; hence, the recurrence rates were 8% (99% CI 3.9–14.2) and 6%, respectively; the *p*-value for non-inferiority was found to be significant <0.01 (Table 3 and Table 4). In addition, both groups had a subjective cure rate higher than 90% for the apical compartment (95% vs. 97% (*p* = 0.655), respectively).

#### 3.1.2. Recurrence, Re-Surgery, and Post-Operative Complications

Regarding the reoperation rate for an apical defect, only five (2.5%) patients in the ALS group and two (2%) in the ASC group required surgery for symptomatic apical recurrence (*p* > 0.999), all via an abdominal approach (Table 3). All of these recurrences were combined apical and anterior or posterior prolapse that were successfully managed with a subsequent sacral approach or lateral suspension strategy based on the primary surgery.

All recurrences in the anterior and posterior compartments are shown in Table 5. The cure rate for the anterior compartment was found similar in both cohorts (83.9% for ALS vs. 80.6% for ASC). Relating the posterior compartment, the two cohorts are not comparable given the different preoperative selection. There was an objective anatomic success for posterior defect in the ASC cohort with an objective cure rate of 85.4%. In the ALS group, at enrollment we excluded patients with an advanced posterior defect. Indeed, only 12 (6%) patients had rectocele (POP-Q ≥ II) and 6 of these enteroceles disappeared after surgery. There were six patients who had a persistence posterior defect and only one required posterior colporrhaphy.

Four patients (three in the ALS group and one in the ASC group) developed an isolated and asymptomatic apical defect due to a cervical elongation that did not require surgical treatment.

None of the patients in either group had a major post-operative complication (Clavien-Dindo grade ≥ 3b) (Table 6).

In the ALS group, five patients reported pain near the anterior superior iliac spine, where the lateral arm of the mesh reaches the abdominal wall (grade 1 and 3a on the Clavien-Dindo scale). Two patients required surgical mesh mobilization through a 1 cm incision posterior to the anterior superior iliac spine, rated grade 3a on the Clavien-Dindo scale. Classified as 2AT2S1O and 2AT3s1, according to the IUGA/ICS Prosthesis/Graft Complication Classification System (Table 6).

In the ASC group, one case of post-operative melena and one sub-fascial hematoma was recorded that required blood transfusion, rated grade 2 on the Clavien-Dindo scale. One patient had an abdominal wall hernia at the level of a 12 mm trocar insertion, rated grade 3a on the Clavien-Dindo scale. Two patients had mesh exposure, classified as grade 2 and 3a on the Clavien-Dindo scale. The latter had a vaginal vault mesh exposure with a pelvic abscess, classified as 3DT3S5, and required vaginal revision and pelvic abscess drainage (Table 6).

Therefore, the mesh complications for ALS and ASC occurred at a rate of 1% and 2%, respectively (Table 6).

## 4. Discussion

To our knowledge, this is the first study to compare lateral suspension (ALS) with sacral suspension (ASC). Our prospective multicenter study shows that ALS is non-inferior to ASC in the treatment of advanced apical prolapse at 12-month follow-up. The objective cure rate for the apical defect was 92% for ALS and 94% for ASC, with a significant *p*-value for non-inferiority < 0.01. Both surgical techniques are effective for apex correction, which is consistent with previous studies of laparoscopic and robotic ALS and ASC [7,19]. The subjective cure rate was higher than 90% for both procedures. No patient had major post-operative complications.

Sacral suspension using minimally invasive techniques remains the gold standard for correction of advanced apical and multi-compartmental prolapse [3]. However, there is a growing interest in alternative surgical strategies to sacral suspension. Accordingly, various surgical techniques have been developed for abdominal suspension. This is not only to minimize the surgical tasks associated with the dissection of the promontory, but also to achieve individualized treatment and patient outcomes.

ALS is a fast procedure that avoids the complex surgical steps of sacrocolpopexy (i.e., sacral and recto-vaginal dissection); thus, it may represent a more convenient approach for isolated apical prolapse or a combined anterior and apical prolapse [20,21].

Since the cornerstone of reconstructive surgery of POP remains the apical defect restoration, the new technique should always be compared to sacrocolpopexy in order to determine the efficacy of apex suspension. The overall anatomical success for ALS reported in the literature is greater than 90% for the apical compartment [22]. Our study confirms this evidence by showing an objective cure rate for apical prolapse of 92%. Furthermore, our study establishes, in a comparative fashion, that this cure rate is non-inferior to the ASC apical repair (92% vs. 94%).

The comparative analysis of the anatomic outcomes of lateral and sacral suspension allows us to speculate that both strategies have strengths and weaknesses in terms of anterior and posterior defect restoration. However, this is not the main focus of our study as the two patient populations have different preoperative anatomical characteristics.

While the two procedures seem to correct similarly apical defect, ASC seems to be effective to treat patients with posterior prolapse who should not be submitted to ALS.

On the contrary, ALS seems to be a highly effective corrective strategy for patients with advanced anterior defect. The anterior cure rate with ALS in our study is 83.9%. The peculiar shape of the mesh allows an effective restoration of the pubo-cervical fascia defect. The lateral traction and the central suspension of the apex is effective in reducing anterior prolapse.

ALS does not correct a posterior defect and it does not confer a posterior displacement of the apex, turning into a larger opening of the Douglas pouch compared to ASC. In line with this concept, we have not submitted patients with advanced posterior defects to ALS.

Our study also sheds light on the processes of apical failure in ALS vs. ASC and the implications for surgical repair. Re-surgery for apical relapse was rare in both groups (2.5% ALS and 2% ASC), and was caused by the mesh detaching from the cervix or vaginal vault in ASC relapse cases or by the lateral arms of the T-shaped mesh downward sliding in ALS cases.

There are no clear protocols on adequately managing an abdominal suspension procedure failure. In this view, the availability of an alternative surgical strategy for apical suspension was critical because it enabled us to manage patients with apical failure using a different technique, suspending them at the sacrum for those who relapsed following ALS and vice versa for those previously treated with ASC. To this extent, lateral suspension is an alternative back-up strategy in case of sacral suspension failure or in case of difficult sacral space dissection (e.g., low caval vein bifurcation).

The incidence of post-operative complications grade ≥3b according to the Claiven-Dindo scale is low in our study. This is consistent with the published literature [19,22]. In terms of the mesh complications, mesh erosion rates for ALS and ASC in the literature ranged from 0% to 13% [23]. This is consistent with the mesh erosion/exposure rate reported in our series, which is 1% and 2% in the ALS and ASC group, respectively. For both procedures, long-term absorbable sutures (polydioxanone) were used to anchor the mesh to the anterior/posterior vaginal wall and non-absorbable 2-0 polypropylene sutures were used to anchor the mesh to the cervix or to the vaginal vault.

Regarding the post-operative pain, only five (2.5%) patients developed pain after surgery; we have ascribed this to a possible nerve entrapment at the level of the anterior-superior iliac spine where the lateral arm of the mesh reaches the abdominal wall. All patients solved the issue with mesh mobilization or medical therapy, and we had no cases of chronic pain.

The difference in the type of mesh used in the two groups introduces a bias into our comparative study. However, the low rate of mesh related complications for both procedures stand in favor of the safety of the abdominal approach.

The main strengths of our study are the large sample size, long follow-up period, and multicenter study design. In addition, our study shows for the first time a comparative analysis of two different abdominal apical POP repairs. On the other hand, the main limitation of the study was the absence of a quality-of-life questionnaire to assess subjective outcomes and a systematic assessment of lower urinary tract symptoms.

## 5. Conclusions

Our prospective multicenter study demonstrates that ALS is a safe, highly effective technique and non-inferior to ASC in the treatment of advanced apical prolapse at 12-months follow-up.

## Figures and Tables

**Table 1:** **Pre-operative demographic characteristics.** Data are presented as n (%) or as median values ± SD. Characteristics comparing ALS vs. ASC before surgery were statistically analyzed with two-tailed Fisher’s exact test or by two-tailed Mann–Whitney test, accordingly. *p*-values < 0.05 were considered statistically significant. ALS: abdominal lateral suspension; ASC: abdominal sacral colpopexy.

Characteristics	ALS (n = 200)	ASC (n = 100)	*p*-value
Age (years), mean (*SD*)	63.1 (8.1)	64.9 (8.8)	0.152
BMI (kg/m^2^), mean (*SD*)	24 (2.4)	24.5 (1.8)	0.163
Multiparous, *n* (%)	197 (98.5%)	95 (95%)	0.122
Vaginal Delivery, *n* (%)	197 (98.5%)	94 (94%)	0.064
Menopausal Status, *n* (%)	169 (84.5%)	92 (92%)	0.072
Hysterectomy Prior Surgery, *n* (%)	7 (3.5%)	34 (34%)	<0.001
Apical Prolapse Stage ≥ II	200 (100%)	100 (100%)	>0.999
Stage II	54 (27%)	15 (15%)	
Stage III	101 (50.5%)	50 (50%)	
Stage IV	45 (22.5%)	35 (35%)	
Anterior Prolapse Stage ≥ II	186 (93%)	72 (72%)	<0.001
Stage II	39 (19.5%)	15 (15%)	
Stage III	94 (47%)	35 (35%)	
Stage IV	53 (26.5%)	22 (22%)	
Posterior Prolapse Stage ≥ II	12 (6%)	55 (55%)	<0.001
Stage II	10 (5%)	24 (24%)	
Stage III	2 (1%)	20 (20%)	
Stage IV	0 (0%)	11 (11%)	

**Table 2:** **Post-operative demographic characteristics.** Data are presented as n (%) or as median values ± SD. Characteristics comparing ALS vs. ASC after surgery were statistically analyzed with two-tailed Fisher’s exact test or by two-tailed Mann–Whitney test, accordingly. *p*-values < 0.05 were considered statistically significant. ALS: abdominal lateral suspension; ASC: abdominal sacral colpopexy.

Characteristics	ALS (n = 200)	ASC (n = 100)	*p*-value
Hysterectomy during surgery, *n* (%)	6 (3%)	33 (33%)	<0.001
Intraoperative Complication, *n* (%)	0 (0%)	1 (1%)	0.110
Operative Time (minutes), mean (SD)	123 (33)	193 (55.6)	<0.001

**Table 3:** **Anatomical outcomes in the apical compartment based on clinical evaluation with a simplified POP-Q measurement as defined by IUGA-ICS prolapse staging.** Age and BMI were used to select 300 patients, with ALS/ASC ratio equal 2, from a population of 360 patients by propensity score method. Data are presented as frequency (%). Simplified POP-Q values comparing ALS vs. ASC before surgery and after surgery were statistically analyzed with a two-tailed Fisher’s exact test. *p*-values < 0.05 were considered statistically significant. POP-Q: Pelvic Organ Prolapse Quantification System; IUGA-ICS: International Urogynecological Association—International Continence Society; ALS: Abdominal Lateral Suspension; ASC: Abdominal Sacral Colpopexy.

Apical Recurrence	ALS (n = 200)	ASC (n = 100)	*p*-value
No	184 (92%)	94 (94%)	0.642
Yes	16 (8%)	6 (6%)	
Re-Surgery	5 (2.5%)	2 (2%)	>0.999

**Table 4:** **Confidence Interval of ALS rate to calculate *p*-value of non-inferiority. Condition of non-inferiority**. Prolapse recurrence variation rate between ASC and ALS not exceeding 10%. *p*-values < 0.05 were considered statistically significant. ALS: Abdominal Lateral Suspension; ASC: Sacral Colpopexy; CI: confidence interval; non-inf: non-inferiority.

Percentage ALS (99% CI)	Percentage ASC	*p*-value (non-inf.)
8 (3.9–14.2)	6 (1.7–14.9)	<0.01

**Table 5:** **Anatomical outcomes in the anterior and posterior compartment based on clinical evaluation with a simplified POP-Q measurement as defined by IUGA-ICS prolapse staging.** Data are presented as frequency (%). Simplified POP-Q values comparing ALS vs. ASC before surgery and after surgery were statistically analyzed with two-tailed Fisher’s exact test. *p*-values < 0.05 were considered statistically significant. POP-Q: Pelvic Organ Prolapse Quantification System; IUGA-ICS: International Urogynecological Association—International Continence Society; ALS: Abdominal Lateral Suspension; ASC: Abdominal Sacral Colpopexy.

Anterior Recurrence	ALS (n = 186)	ASC (n = 72)	*p*-value
no	156 (83.9%)	58 (80.6%)	0.580
yes	30 (16.1%)	14 (19.4%)	
Repeat Surgery	4 (2.1%)	4 (5.5)	0.242
**Posterior Recurrence**	**ALS (n = 12)**	**ASC (n = 55)**	***p*-value**
no	6 (50%)	47 (85.4%)	0.013
yes	6 (50%)	8 (14.6%)	
Repeat Surgery	1 (8.3%)	0 (0%)	0.429

**Table 6:** **Post-operative complications.** Data are presented as n (%). Characteristics comparing ALS vs. ASC after surgery were statistically analyzed with two-tailed Fisher’s exact. *p*-values < 0.05 were considered statistically significant. ALS: Abdominal Lateral Suspension; ASC: Abdominal Sacral Colpopexy.

Characteristic	ALS (n = 200)	ASC (n = 100)	*p*-value
Clavien-Dindo < 30 days			0.476
Grade 1	10 (5.0%)	3 (3.0%)	
Grade 2	2 (1.0%)	3 (3.0%)	
Grade 3a	1 (0.5%)	1 (1.0%)	
Grade 3b	0 (0.0%)	0 (0.0%)	
Grade 4	0 (0.0%)	0 (0.0%)	
Grade 5	0 (0.0%)	0 (0.0%)	
Clavien-Dindo > 30 days			0.472
Grade 1	12 (6.0%)	0 (0.0%)	
Grade 2	0 (0.0%)	2 (2.0%)	
Grade 3a	4 (2.0%)	3 (3.0%)	
Grade 3b	0 (0.0%)	0 (0.0%)	
Grade 4	0 (0.0%)	0 (0.0%)	
Grade 5	0 (0.0%)	0 (0.0%)	

## Data Availability

All the data used to support the findings of this study are included in the article.
